# Patient-derived organoids in non-small cell lung cancer: advances in drug sensitivity testing

**DOI:** 10.3389/fphar.2025.1639268

**Published:** 2026-01-05

**Authors:** Wanyu Tang, Xudong Tian

**Affiliations:** Department of Health Medicine, Chongqing Youth Vocational and Technical College, Chongqing, China

**Keywords:** lung cancer, organoids, tumor microenvironment, high-throughput drug screening, drug resistance, precision therapy

## Abstract

Patient-derived organoids (PDOs) have emerged as transformative preclinical models in non-small cell lung cancer (NSCLC), offering high-fidelity recapitulation of tumor heterogeneity and drug responses. Compared to traditional cell lines and xenografts, PDOs preserve the genetic, phenotypic, and functional features of parental tumors, enabling precise drug sensitivity testing for chemotherapy, targeted therapy, and immunotherapy, particularly through optimized culture protocols, genetic engineering techniques, and cryopreservation methods, have significantly enhanced their scalability and clinical relevance. PDOs have proven instrumental in elucidating key resistance mechanisms such as EGFR-TKI resistance mediated through DCLK1-dependent Wnt signaling activation, while simultaneously identifying novel therapeutic synergies for clinical translatio. However, challenges remain in modeling the tumor immune microenvironment and standardizing clinical translation. This review systematically outlines the advancements and challenges in establishing NSCLC PDOs, highlights the potential of PDOs to guide personalized NSCLC therapy while addressing current limitations to bridge the gap between research and clinical application.

## 1 Introduction

Lung cancer remains one of the most prevalent and lethal malignancies worldwide (Dijkstra et al., 2025). This poor prognosis is largely attributable to the genomic instability of tumor cells, which leads to both germline and somatic mutations, thereby fostering pronounced inter- and intra-tumoral heterogeneity and therapeutic resistance (Canale et al., 2025; Nousias et al., 2025; Kobay et al., 2023). The identification of suitable experimental models for drug sensitivity screening represents a promising strategy for precision therapy. Beyond empiric treatment paradigms, individualized drug susceptibility testing may enable the selection of optimal therapeutic agents, thereby improving clinical outcomes. Traditional anti-cancer drug screening systems primarily rely on patient-derived tumor cell (PDC) models and patient-derived xenograft (PDX) models. The PDC model offers several advantages, including rapid proliferation, low maintenance cost, and amenability to functional assays, and has thus been widely employed in high-throughput drug screening platforms (Xu et al., 2022; Ma et al., 2022a). However, PDCs fail to faithfully recapitulate the phenotypic and genetic complexities of *in vivo* tumors and cannot accurately model the dynamic progression and heterogeneity of cancer. In contrast, PDX models preserve the tumor microenvironment, histopathological architecture, and genomic landscape of the parental tumor, rendering them valuable tools for anti-cancer drug screening, and for predicting therapeutic efficacy, toxicity, adverse events, and drug bioavailability. Nonetheless, the generation of PDX models is dependent on immunocompromised murine hosts, which differ significantly from humans in both genetic and biological contexts (LeSavage et al., 2022).

Patient-derived organoids (PDOs) represent a novel class of 3D miniature tumor models derived from primary tumor tissues harvested directly from patients (Wang et al., 2022; Li and Izpisua Belmonte, 2019). These models retain the essential histopathological, genetic, and phenotypic attributes of the parental tumors. Compared to PDC and PDX models, PDOs can be established within microenvironments that more closely approximate physiological conditions, thereby supporting faithful phenotypic replication and robust tumor cell proliferation (Drost and Clevers, 2018; Rossi et al., 2022). Given their high fidelity in recapitulating the histological and functional characteristics of the original tumors, PDOs have found broad applications in regenerative medicine and mechanistic disease research. In the context of lung cancer, PDOs serve as innovative *ex vivo* models capable of recapitulating the biological features of primary tumors with high precision. When co-cultured with extracellular matrix components and stromal elements, they further enable the reconstruction of the tumor microenvironment (Li and Izpisua Belmonte, 2019). These attributes make lung cancer PDOs a potentially powerful tool for drug sensitivity testing and for advancing personalized treatment strategies. Although the utility of lung cancer patient-derived organoids (PDOs) has been reviewed from multiple perspectives (Navarro et al., 2025), systematic and in-depth evaluations of their role in guiding individualized therapy remain limited. While preliminary studies demonstrate that lung cancer PDOs show promise in drug sensitivity assays for clinical decision-making (Adhikary et al., 2025), most investigations have assessed only a narrow spectrum of therapeutic agents. This review highlights the innovative advantages of lung cancer PDOs, focusing specifically on recent advances in their application as drug sensitivity testing platforms for conventional chemotherapeutics, targeted agents, and immunotherapies. Furthermore, this review outlines the current limitations to provide a balanced perspective on the potential and challenges of PDOs in lung cancer treatment.

## 2 Establishment and optimization of NSCLC organoid models

### 2.1 Technical challenges in organoid derivation

NSCLC organoids are predominantly established from surgical tumor specimens (Ma J. et al., 2025). Typically, tissues are mechanically dissociated into small fragments and digested enzymatically using collagenase or neutral protease at 37 °C to yield single cells or small clusters, which are subsequently cultured to form NSCLC organoids (Kim and Park, 2025). To improve tumor cell purity and better recapitulate tumor heterogeneity, Li Z. et al. (2021) optimized the use of minimally invasive biopsy specimens or malignant pleural effusions, reducing contamination from normal pulmonary cells. The efficiency of tumor cell isolation depends critically on enzymatic digestion parameters, including enzyme type, concentration, digestion mode, and duration, which influence both cell yield and viability. To mitigate enzymatic damage to tumor cells, preserve tissue integrity, and streamline cell isolation, Tamura et al. (2018) and Takahashi et al. (2019) developed a method using mechanically minced tumor tissue for suspension culture. After stromal depletion, prolonged culture (3–6 months) in matrix-free medium yielded a biobank of 15 NSCLC organoids. Li Z. et al. (2021), Li Z. et al. (2020) observed that some NSCLC organoids exhibit limited proliferative capacity after serial passaging, regardless of enzymatic conditions. Alternative protocols (Li et al., 2018; Sachs et al., 2019) addressed this by employing mechanical dissociation during passaging, fragmenting organoids without enzymatic treatment, thereby improving long-term expansion. Further refinements in organoid handling have enhanced practicality. Kim et al. (2019) identified organoid diameter (100–150 μm) and storage temperature (4 °C) as critical factors for cryopreservation, achieving 70% post-thaw recovery via low-speed centrifugation and liquid nitrogen storage. Compared to 2D cultures, the 3D complexity of PDOs introduces additional challenges. To streamline culture conditions, Zhang et al. (2016) designed a superhydrophobic microwell array chip that isolates organoids using hydrophobic microstructures. This platform simplifies medium exchange, improves viability, and increases culture success rates by reducing technical variability.

Another critical challenge in the establishment of NSCLC organoids is the maintenance of genetic stability over prolonged culture periods (Li Z. et al., 2024). As organoids undergo long-term expansion, genetic drift that manifested as the accumulation of somatic mutations and chromosomal aberrations can occur, potentially altering the tumor’s genomic landscape (Nam et al., 2022). This genetic instability can profoundly impact drug response data by introducing mutations that confer resistance to certain therapies or enhance sensitivity to others (Broutier et al., 2017). For instance, mutations in the EGFR signaling pathway, such as the acquisition of the T790M mutation, could render organoids resistant to EGFR inhibitors, despite initial sensitivity (Lim et al., 2024). Similarly, mutations in KRAS, particularly G12C, could alter the tumor’s response to targeted therapies like EGFR-TKIs and MEK inhibitors by activating downstream MAPK and PI3K/AKT/mTOR pathways that promote tumor survival and drug resistance (Song et al., 2024; Ma X. et al., 2025). Genetic drift may also activate the Wnt/β-catenin pathway, which regulates tumor progression and stem cell maintenance, further contributing to therapy resistance (Yan et al., 2022). Additionally, ferroptosis, a regulated form of cell death suppressed by genetic alterations such as GPX4 loss (Liao et al., 2024), may be dysregulated in PDOs, particularly those exposed to chemotherapy, leading to enhanced resistance. These alterations, driven by genetic drift, underscore the potential for PDOs to evolve differently from the original tumor, complicating the predictive value of drug response assays. Therefore, long-term culture-induced genetic drift must be carefully considered when interpreting PDO-based drug sensitivity data.

### 2.2 Current frontiers in NSCLC organoid systems

A critical challenge in establishing NSCLC organoids from surgical specimens is the overgrowth of normal cells, which reduces tumor purity (Lee et al., 2021). To address this, Kumamot et al. (2008) found that nutlin-3a supplementation selectively eliminates non-tumor cells, promoting the expansion of TP53-mutant organoids. However, prolonged use of such agents may induce genomic instability and alter tumor-specific characteristics (Kucab et al., 2017). Alternative approaches, such as embedding dissociated tumor cells in matrigel with lung epithelial-optimized media, have proven more effective in suppressing normal cell growth while preserving cancer genomic integrity (Kim et al., 2019; Shi et al., 2020). Recent advances focus on leveraging organoids for personalized therapy (Han et al., 2022; Kim SK. et al., 2021). For instance, Banda et al. (2020) cultured organoids in erlotinib-containing matrigel, monitoring genomic evolution and resistance mechanisms. Their findings recapitulated acquired resistance in NSCLC, providing insights for therapeutic optimization. Beyond primary tumor-derived models, genetically engineered normal organoids offer a controlled system to study carcinogenesis. Naranjo et al. (2022) induced oncogenic mutations in normal lung organoids, triggering malignant transformation and tumor formation upon murine transplantation. Similarly, Dost et al. (2020) generated KRAS-mutant organoids, which exhibited diminished epithelial biomarker expression, validating their use in early-stage cancer modeling. While 3D matrigel-based systems (Shi et al., 2020) facilitate tumor-specific studies, their lack of native stromal and immune components restricts microenvironmental recapitulation (Yuki et al., 2020). Consequently, co-culture models integrating tumor organoids and immune cells are emerging as a vital tool to investigate tumor-immune crosstalk, potentially advancing NSCLC immunotherapy research (Dijkstra et al., 2018; Nakamura et al., 2019).

## 3 Applications of NSCLC organoids in drug screening

Organoid models have emerged as a transformative tool for recapitulating critical features of tumor biology. As patient-derived systems, they maintain the genomic and histopathological characteristics of primary tumors during long-term culture, making them invaluable for large-scale drug discovery. A key advancement was achieved by Takahashi et al. (2019), who developed a high-throughput screening platform using 96- or 384-well plates. Their innovative use of CellPetFT filters enabled uniform organoid fragmentation while preserving spheroid integrity, overcoming a major bottleneck in organoid-based assays and enabling scalable pharmacological testing. Further enhancing their utility, Liu et al. (2021) demonstrated that cryopreservation via a superhydrophobic micropore array chip system maintained identical drug sensitivity profiles in organoids post-thaw. These methodological breakthroughs have accelerated the integration of organoids into preclinical pharmacology, where they now play a pivotal role in studying therapeutic response and resistance mechanisms (Banda et al., 2020; Liu et al., 2021; Chen et al., 2022; Lin et al., 2021).

### 3.1 PDOs recapitulate NSCLC pathobiology and drug responses

#### 3.1.1 PDOs model NSCLC heterogeneity and drug response

Compared with traditional PDC and PDX models, PDOs better recapitulate tumor heterogeneity and drug response patterns. Kim et al. (2019) established organoids harboring specific oncogenic mutations, which responded predictably to targeted therapies. For instance, BRCA2-mutant organoids showed sensitivity to olaparib, EGFR-mutant organoids responded to erlotinib, and organoids with EGFR mutations coupled with MET amplification exhibited high sensitivity to crizotinib (Levantini et al., 2022; Momozawa et al., 2022; Remon et al., 2023). These findings confirm that transcriptomic signatures in PDOs can serve as reliable indicators for drug sensitivity profiling. Kim SY. et al. (2021) evaluated the efficacy of dabrafenib/trametinib combination therapy using organoids simultaneously harboring EGFR exon 19 deletion, BRAF mutations, and the BRAF G464A variant, validating the therapeutic synergy (Sforza et al., 2022). Subsequent investigations (Li et al., 2022; Rosen et al., 2022) systematically evaluated targeted agents, including poziotinib for ERBB2 exon 20 insertions, pralsetinib for kinase fusions, and afatinib for EGFR L747P mutations—revealing strong correlation between organoid responses and clinical efficacy. Mechanistically, KRAS-mutant NSCLC organoids recapitulated the clinical resistance profile to erlotinib/gefitinib, while afatinib/neratinib overcame this by modulating ERBB2/3 signaling (Papadimitrakopoulou et al., 2016; Rulli et al., 2015). Chen et al. (2020), conducted a comprehensive validation, testing 50 PDO lines against 26 NCCN-recommended agents. Their findings precisely mirrored clinical observations: EGFR exon 20 insertion organoids responded to osimertinib/chemotherapy but resisted gefitinib, c-MET-overexpressing models were osimertinib-sensitive but gefitinib-resistant, and KRAS G12C mutants showed enhanced TKI response with ERBB pathway inhibition, systematically validating PDOs as predictive platforms for precision oncology.

#### 3.1.2 PDOs bridge mechanistic and target discovery in NSCLC

PDOs serve as versatile platforms for both elucidating drug mechanisms and identifying novel therapeutic targets. Ji et al. (2023) demonstrated that NRF2 promotes lung squamous cell carcinoma growth via PI3K-Akt-mTOR signaling, with NRF2 inhibition effectively suppressing this pathway. Similarly, Taverna et al. (2020) utilized short-term lung adenocarcinoma organoids to reveal compensatory crosstalk between AXL and JAK-STAT pathways upon targeted inhibition. These mechanistic insights are complemented by PDOs’ utility in target discovery: β5-integrin was found to suppress gastrin-mediated ferroptosis (Tan et al., 2021), while Su et al. (2023) showed that Src or ceramidase inhibition could reactivate ferroptosis in resistant tumors. Furthermore, Pan et al. (2023) identified Kmt2d deletion as a driver of RTK-RAS signaling through EGFR/ERBB2 upregulation, with combined SHP2 and afatinib inhibition demonstrating potent antitumor effects. Together, these studies highlight PDOs’ dual role in both mechanistic investigation and therapeutic target identification.

### 3.2 PDOs advance resistance research and NSCLC therapy

**FIGURE 1:**
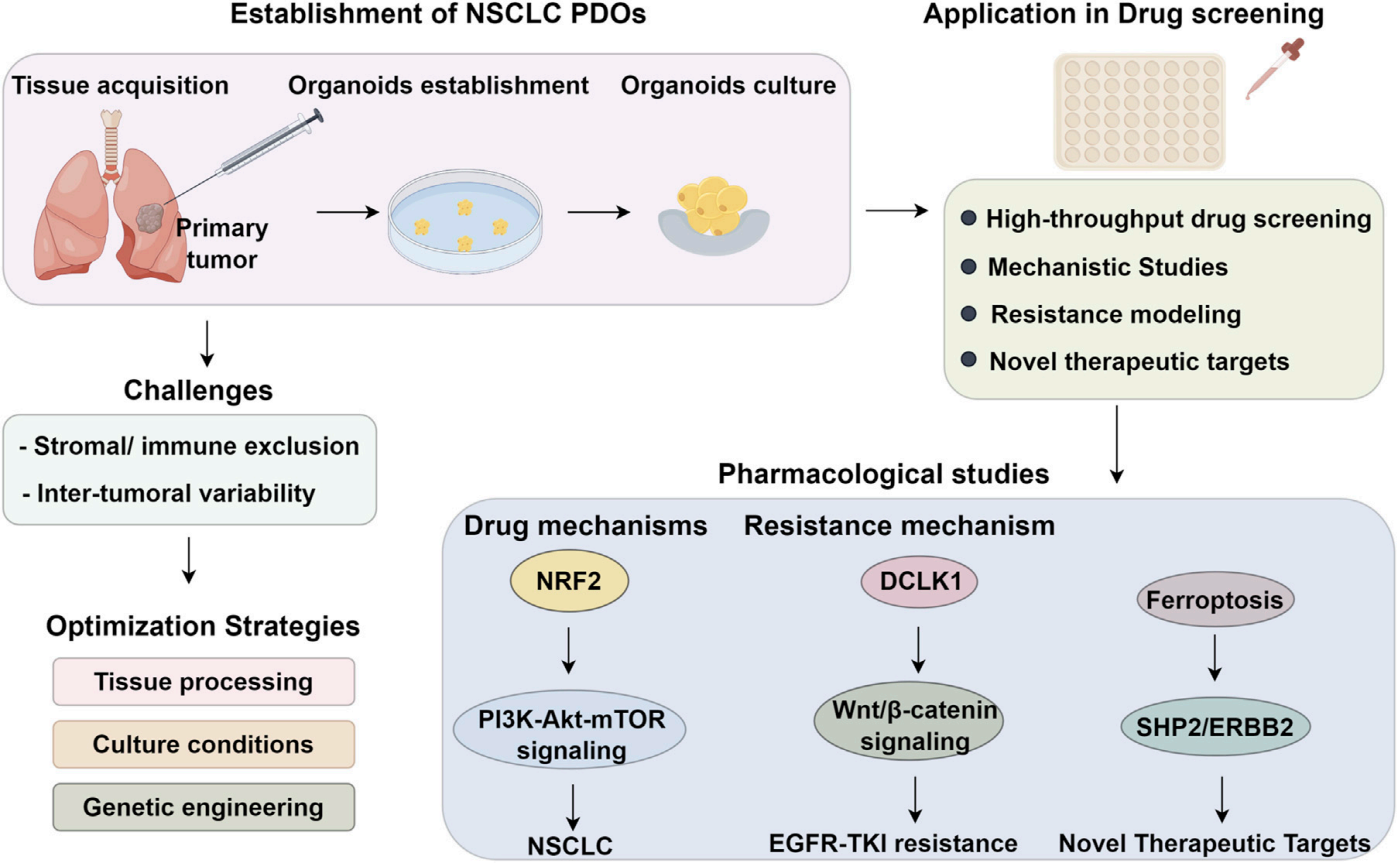
Workflow and translational application of PDOs in NSCLC. Schematic overview depicting the establishment, optimization, and application of patient-derived organoids (PDOs) in non-small cell lung cancer (NSCLC). Primary tumor tissues obtained from surgical or biopsy specimens undergo dissociation and are cultured under 3D conditions to generate NSCLC PDOs. Key challenges include the exclusion of stromal and immune components and inter-tumoral variability, which limit microenvironmental fidelity and clinical relevance. Optimization strategies such as refined tissue processing, tailored culture conditions, and genetic engineering approaches have improved PDO viability and scalability. These models are subsequently utilized in high-throughput drug screening platforms to assess therapeutic efficacy, explore pharmacodynamic mechanisms, model resistance phenotypes, and identify novel therapeutic targets. Mechanistic studies have highlighted the role of NRF2-driven PI3K–Akt–mTOR signaling in tumor growth, while resistance modeling has uncovered DCLK1-mediated Wnt/β-catenin activation and ferroptosis suppression as contributors to EGFR-TKI resistance. The identification of SHP2/ERBB2 axis vulnerabilities further illustrates the potential of PDOs to guide precision oncology in NSCLC.

PDOs demonstrate significant potential in predicting patient-specific drug responses and elucidating resistance mechanisms. Li YF. et al. (2020) evaluated five natural compounds in PDOs, revealing distinct response patterns: berberine exhibited selective efficacy in PDOs but not in conventional cell lines, while betaine induced resistance in both models. Notably, PDOs also enable real-time monitoring of acquired resistance. Halofuginone (HF) was shown to suppress proliferation, induce cell cycle arrest, and restore cisplatin sensitivity in resistant PDOs, confirming its dual role as a chemosensitizer and pathway inhibitor (Li H. et al., 2021). Further mechanistic studies identified recombinant DCLK1 as a key regulator of cancer stemness via Wnt/β-catenin signaling, driving EGFR-TKI resistance. Yan et al. (2022) demonstrated that DCLK1 inhibition restored TKI sensitivity and synergized with EGFR inhibitors in TKI-resistant PDOs. These findings highlight the role of DCLK1 in promoting stemness and its potential as a therapeutic target in overcoming resistance. Additionally, Weng et al. (2023) developed a novel topoisomerase I inhibitor with potent activity against refractory EGFR-mutant organoids, including those with ex19del/T790M/C797S mutations, and demonstrated synergistic effects with PARP/ATR inhibitors and immunotherapy. These findings underscore PDOs’ utility in identifying drug-tolerant persister (DTP) cells and developing strategies to overcome therapeutic resistance (Mikubo et al., 2021) (Figure 1).

Resistance mechanisms associated with cancer stem cells are critical for sustained tumor growth and survival. In particular, drug-tolerant persister (DTP) cells evade chemotherapies and targeted agents (Li et al., 2023a). MAPK inhibitors induce PINK1-mediated mitophagy in DTP cells, promoting their survival and proliferation (Li et al., 2023b). When combined with chloroquine, an autophagy inhibitor, MAPK inhibitors regain their cytotoxic effects, indicating that targeting mitophagy could offer a strategy to overcome resistance in DTP populations (Li et al., 2023b; Wang B. et al., 2023). Metabolic alterations also play a significant role in resistance to cancer therapies (Lin et al., 2023). One such example is ferroptosis, a form of regulated necrosis. Ferroptosis suppression by β5-integrin has been linked to chemotherapy resistance in PDOs. SRC or ceramidase inhibition reactivated ferroptosis in chemotherapy-resistant PDOs, thereby reversing drug resistance and providing a potential therapeutic avenue for targeting metabolic escape mechanisms in resistant NSCLC tumors (Beekhof et al., 2023; Luk et al., 2024). Despite these advancements, tumor organoids are not yet clinically approved for guiding therapy. Only when organoids exhibit drug responses consistent with matched patients can they be reliably used in personalized medicine (Xiang et al., 2024). Additionally, tumor heterogeneity complicates the evaluation of organoid predictive value. Thus, while promising, organoid-based drug screening remains a substantial challenge requiring further optimization (Table 1).

**TABLE 1:** Pharmacological and translational applications of NSCLC PDOs.

Application area	PDO-based strategy	Targets	Efficiency
Targeted therapy response	Mutation-guided drug sensitivity testing	EGFR-mutant: Erlotinib; BRCA2: Olaparib; MET amplification: Crizotinib	High concordance with patient outcomes
Drug combination validation	Multi-mutation organoids tested with drug cocktails	BRAF G464A+ EGFR ex19del: Dabrafenib + trametinib	Personalized combination regimens
Resistance mechanism analysis	Evaluation of acquired resistance and bypass pathways	EGFR-TKI resistance via DCLK1/Wnt/β-catenin; ferroptosis suppression	PDOs replicate real-time resistance evolution
Sensitizer discovery	Co-treatment assays in resistant PDOs	Halofuginone restores cisplatin sensitivity	Novel adjuvant strategies for chemoresistance
Pathway inhibition studies	Genetic and pharmacologic manipulation in PDOs	NRF2–PI3K–mTOR; AXL–JAK1–STAT3; ERBB2/3–MAPK	Target validation and inhibitor prioritization
Novel therapeutic targets	SHP2 inhibition in Kmt2d-deficient PDOs	SHP099 + afatinib synergize in EGFR/ERBB2-high tumors	Exploiting epigenetic vulnerabilities
High-throughput screening	96/384-Well plate PDO platforms with automated viability readouts	Poziotinib, afatinib, pralsetinibetc.	Enables scalable pharmacotyping for drug discovery

### 3.3 Next-gen PDOs in immunotherapy response prediction

While patient-derived organoids (PDOs) are invaluable in drug screening, their utility in immunotherapy testing remains limited due to the absence of key immune components that are critical for assessing immune-related responses (Jeong and Kang, 2023; Wang M. et al., 2025). This limitation restricts the ability of PDOs to accurately model the immune microenvironment of tumors, which plays a significant role in the efficacy of immunotherapies, such as immune checkpoint inhibitors (Teijeira et al., 2022). To address this limitation, recent studies have established advanced co-culture platforms that incorporate immune components into PDOs, thereby reconstructing a more immunocompetent tumor microenvironment (Magré et al., 2023; Dao et al., 2022). Immune cells such as peripheral blood mononuclear cells (PBMCs), tumor-infiltrating lymphocytes (TILs), dendritic cells, macrophages, and even chimeric antigen receptor T cells (CAR-T cells) have been successfully integrated into NSCLC PDO systems. These immune cells can be derived either autologously from patient or from allogeneic sources (Sun et al., 2022; Li P. et al., 2024). Co-culture techniques vary, including embedding immune cells into Matrigel with PDOs, overlaying immune cells on organoid surfaces, and using organoid-on-a-chip models to preserve spatial architecture and facilitate direct immune-tumor interaction (Dao et al., 2022; Fang et al., 2023). To overcome this challenge, recent studies have developed co-culture systems that integrate immune cells with PDOs, thus allowing for a more accurate representation of the tumor-immune interaction. These tumor–immune organoid platforms have been used to evaluate immune checkpoint inhibitors, such as PD-1/PD-L1 and CTLA-4 antibodies, providing insights into their efficacy in a more physiologically relevant context (Amato et al., 2025; Ou et al., 2022). For example, Hélène et al. co-cultured NSCLC PDOs with IFN-γ, which led to enhanced immune response assessments and a better understanding of the tumor’s immune evasion mechanisms (Lê et al., 2024). Furthermore, PDO-based immune checkpoint evaluation systems have also been introduced, where immune cells are incorporated into PDO cultures to assess how tumors interact with immune cells and respond to checkpoint inhibition (Yao et al., 2025).

In addition to immune components, the incorporation of other stromal cell types, particularly endothelial cells, has gained attention for reconstructing a more comprehensive tumor microenvironment within PDO systems (Madan et al., 2025; Podaza et al., 2022). Endothelial cells play a pivotal role in angiogenesis, tumor perfusion, and immune cell trafficking, all of which influence drug delivery and response (Xu et al., 2024; Zhao et al., 2021). Advanced organoid-on-a-chip platforms and microfluidic co-culture systems now allow the inclusion of human endothelial cells alongside PDOs, enabling the formation of rudimentary vascular networks within the extracellular matrix (Zhao et al., 2021; Lim et al., 2021). These vascularized organoid models better replicate *in vivo* gradients of oxygen and nutrients, facilitate dynamic cell–cell interactions, and improve drug penetration modeling. For example, co-culturing NSCLC PDOs with human umbilical vein endothelial cells (HUVECs) under perfusion flow conditions has been shown to support neovascular structure formation and recapitulate tumor angiogenic responses to VEGF inhibitors (Ma et al., 2022b). Additionally, incorporation of pericyte-like stromal cells further enhances the maturation and stability of these networks (Wang X. et al., 2023; Phan et al., 2017).

Notably, studies utilizing TIL-PDO co-cultures have demonstrated that cytotoxic CD8^+^ T cells infiltrate the organoid structure and mediate cell killing in response to PD-1 blockade, thereby validating the translational potential of such systems (Dijkstra et al., 2018; Magré et al., 2023). Similarly, ALI-PDO platforms preserve endogenous immune infiltrates within the PDO structure, offering a superior representation of *in situ* immunity (Neal et al., 2018; Thorel et al., 2024). Furthermore, time-lapse imaging and cytokine release assays (IFN-γ, Granzyme B) have been used to functionally assess immune activation and killing capacity (Yuki et al., 2020; Gu et al., 2024). To further enhance the physiological relevance of PDOs and enable dynamic modeling of tumor microenvironments, microfluidic platforms will be an important component of next-generation organoid systems (Szewczyk et al., 2024; Sontheimer-Phelps et al., 2019). Notably, microfluidic devices allow precise spatial and temporal control over cell–cell and cell–matrix interactions (Bhatia and Ingber, 2014; DePalma et al., 2025), which are essential for simulating immune infiltration, angiogenesis, and metastatic dissemination within PDO models (Wang J. et al., 2025). These innovative approaches hold the potential to significantly improve the predictive accuracy of PDOs for immunotherapy responses and guide the development of personalized immunotherapy strategies.

## 4 Conclusion

Patient-derived organoids represent a paradigm shift in NSCLC research, combining the physiological relevance of *in vivo* models with the tractability of *in vitro* systems. This review underscores their superiority over traditional PDC and PDX models in preserving tumor heterogeneity and predicting patient-specific drug responses, as evidenced by high concordance between organoid data and clinical outcomes. Key advancements in organoid generation, particularly through optimized enzymatic digestion, cryopreservation, and engineered mutagenesis techniques, have addressed early challenges in scalability and purity.

Furthermore, PDOs have proven invaluable in dissecting resistance mechanisms and identifying synergistic therapies. For instance, they have elucidated EGFR-TKI tolerance mediated through DCLK1-dependent Wnt signaling activation and revealed effective combination strategies such as SHP2/ERBB inhibition for Kmt2d-deficient tumors. However, critical gaps remain, such as the lack of immune-microenvironment recapitulation and standardized validation protocols for clinical translation. Future efforts must prioritize co-culture systems integrating stromal/immune cells, multicenter biobanking initiatives, and prospective clinical trials correlating PDO-guided therapy with patient survival. As the field evolves, PDOs hold immense promise to revolutionize precision oncology, provided these challenges are systematically addressed to ensure reproducibility and clinical utility.
